# *Brucella* mediates autophagy, inflammation, and apoptosis to escape host killing

**DOI:** 10.3389/fcimb.2024.1408407

**Published:** 2024-10-23

**Authors:** Yaqiong Qin, Gengxu Zhou, Fengyuan Jiao, Chuan Cheng, Chi Meng, Lingjie Wang, Shengping Wu, Cailiang Fan, Jixiang Li, Bo Zhou, Yuefeng Chu, Hanwei Jiao

**Affiliations:** ^1^ The College of Veterinary Medicine, Southwest University, Chongqing, China; ^2^ Animal Epidemic Prevention and Control Center of Rongchang, Chongqing, China; ^3^ Veterinary Research Institute, Chinese Academy of Agricultural Sciences, Changchun, Jilin, China; ^4^ State Key Laboratory for Animal Disease Control and Prevention, College of Veterinary Medicine, Lanzhou University, Lanzhou Veterinary Research Institute, Chinese Academy of Agricultural Sciences, Lanzhou, Gansu, China

**Keywords:** *Brucella*, intracellular survival, autophagy, inflammation, apoptosis

## Abstract

Brucellosis is a serious zoonosis caused by *Brucella* spp. infection, which not only seriously jeopardizes the health of humans and mammals, but also causes huge economic losses to the livestock industry. *Brucella* is a Gram-negative intracellular bacterium that relies primarily on its virulence factors and a variety of evolved survival strategies to replicate and proliferate within cells. Currently, the mechanisms of autophagy, inflammation, and apoptosis in *Brucella*-infected hosts are not fully understood and require further research and discussion. This review focuses on the relationship between *Brucella* and autophagy, inflammation, and apoptosis to provide the scientific basis for revealing the pathogenesis of *Brucella*.

## 1 Introduction

Brucellosis is a prevalent, chronic infectious disease caused by the bacterium *Brucella*. So far, twelve *Brucella* species have been isolated and identified. There are six classical species: *Brucella melitensis* (*B. melitensis*), *Brucella abortus* (*B. abortus*), *Brucella suis* (*B. suis*), *Brucella ovis* (*B. ovis*), *Brucella canis* (*B. canis*) and *Brucella neotoma* (*B. neotoma*) (Roop et al., 2021). The species show a high degree of similarity at the genetic level but differ in host preference, zoonotic risk, and virulence (Suárez-Esquivel et al., 2020). *B. melitensis*, *B. abortus*, *B. suis*, *B. canis*, and *B. ovis* are some of the more well-studied *Brucella* species that have sickened goats and sheep, cattle, pigs, dogs, and sheep, respectively (Roop et al., 2021). Animals infected with *Brucella* can cause abortion, infertility, and reduced productivity. *B. melitensis*, *B. abortus*, *B. suis*, and *B. canis* are pathogenic to humans. *B. melitensis* is the most virulent to humans, followed by *B. suis*, and the weakest is *B. abortus* (Deng et al., 2019). People often become ill through direct contact with tissues or blood from diseased animals or through accidental ingestion of products (e.g., dairy products) from infected animals. The common symptoms include undulant fever, sweating, fatigue, anorexia, and joint pain (de Figueiredo et al., 2015a).

*Brucella* has evolved various survival strategies to escape killing by host cells, such as stimulating autophagy after invasion of the host, forming autophagosome-like structures, and preventing lysosomal fusion to allow proliferation, as have *Legionella pneumophila* and *Porphyromonas gingivalis* (Dorn et al., 2002). Autophagy is an innate immune mechanism of the host cell that maintains cellular homeostasis by removing damaged proteins and organelles from the host cell. Some pathogens have evolved strategies that in turn utilize autophagy mechanisms to survive inside cells. T4SS is an important virulence factor of *Brucella*, which secretes effectors such as RicA, VceA, VecC, BtpA, BtpB, BspJ, BspG, BspA, BspB, BspC, BspE, BspF, BPE005, BPE123, BPE043, BPE275, and SepA. VceA and BtpB can regulate autophagy (Zhang et al., 2019; Li et al., 2022). Several autophagy proteins are involved in intracellular replication in *Brucella*: WIPI and ATG9 are involved in the formation of replicative *Brucella*-containing vacuole (rBCV) (Taguchi et al., 2015), and Beclin1 and ATG14L are involved in the formation of autophagic BCV (aBCV) (Starr et al., 2012). When pathogenic microorganisms infect the organism, host cells may promote the maturation and secretion of certain inflammatory factors that initiate natural immune responses and inflammation, contributing to the host’s immune defense against the pathogen. We discussed several pathways of inflammatory response triggered by *Brucella* invasion of the host: VceC triggers an inflammatory response by inducing an unfolded protein response (UPR) (de Jong et al., 2013; Keestra-Gounder et al., 2016); Inflammatory responses triggered by the inflammasomes NLRP3 and AIM2 (Gomes et al., 2013; Costa Franco et al., 2019); Other pathogen-associated molecular patterns (PAMPs) of *Brucella*, such as LPS, major outer membrane proteins, and lipoproteins, trigger inflammatory responses via the TLR pathway. At the same time, *Brucella* uses virulence factors (e.g., BtpA, BtpB, Omp25, etc.) to hinder pro-inflammatory signaling pathways and evade host innate immunity. Apoptosis, also known as programmed cell death, is an autonomous, orderly death of the organism that occurs under genetic regulation. Strongly virulent *Mycobacterium tuberculosis* inhibits apoptosis and weakly virulent *Mycobacterium tuberculosis* promotes apoptosis (Zhai et al., 2019). Similar to *M. tuberculosis*, *Brucella* can also promote or inhibit apoptosis under different conditions and establish a persistent infection by regulating apoptosis. *Brucella* can infect professional phagocytes as well as non-professional phagocytes. Inhibition of apoptosis in infected cells seems to be beneficial to *Brucella* (de Figueiredo et al., 2015b), but there is no definitive answer to date. Some virulence factors of *Brucella* have pro- or inhibitory effects on apoptosis. Omp25 and Omp31 are the major outer membrane proteins of *Brucella*. BspJ, BspG, BspF, Omp25, and Omp31 inhibit apoptosis (Ma et al., 2015; Zhang et al., 2016; Ma et al., 2020; Ma et al., 2022a; Lin et al., 2023). VceA and BtpB promote apoptosis (Zhang et al., 2019; Li et al., 2022). VceC inhibits or promotes apoptosis through different pathways (Byndloss et al., 2019; Zhi et al., 2019).

The mechanisms of autophagy, inflammation, and apoptosis in *Brucella*-infected hosts have not been fully and systematically elucidated. This paper focuses on the relationship between *Brucella* and autophagy, inflammation, and apoptosis, laying the foundation for further unraveling the pathogenic mechanism of *Brucella*.

## 2 Pathogenesis of *Brucella*

### 2.1 Intracellular transport

**Figure 1:**
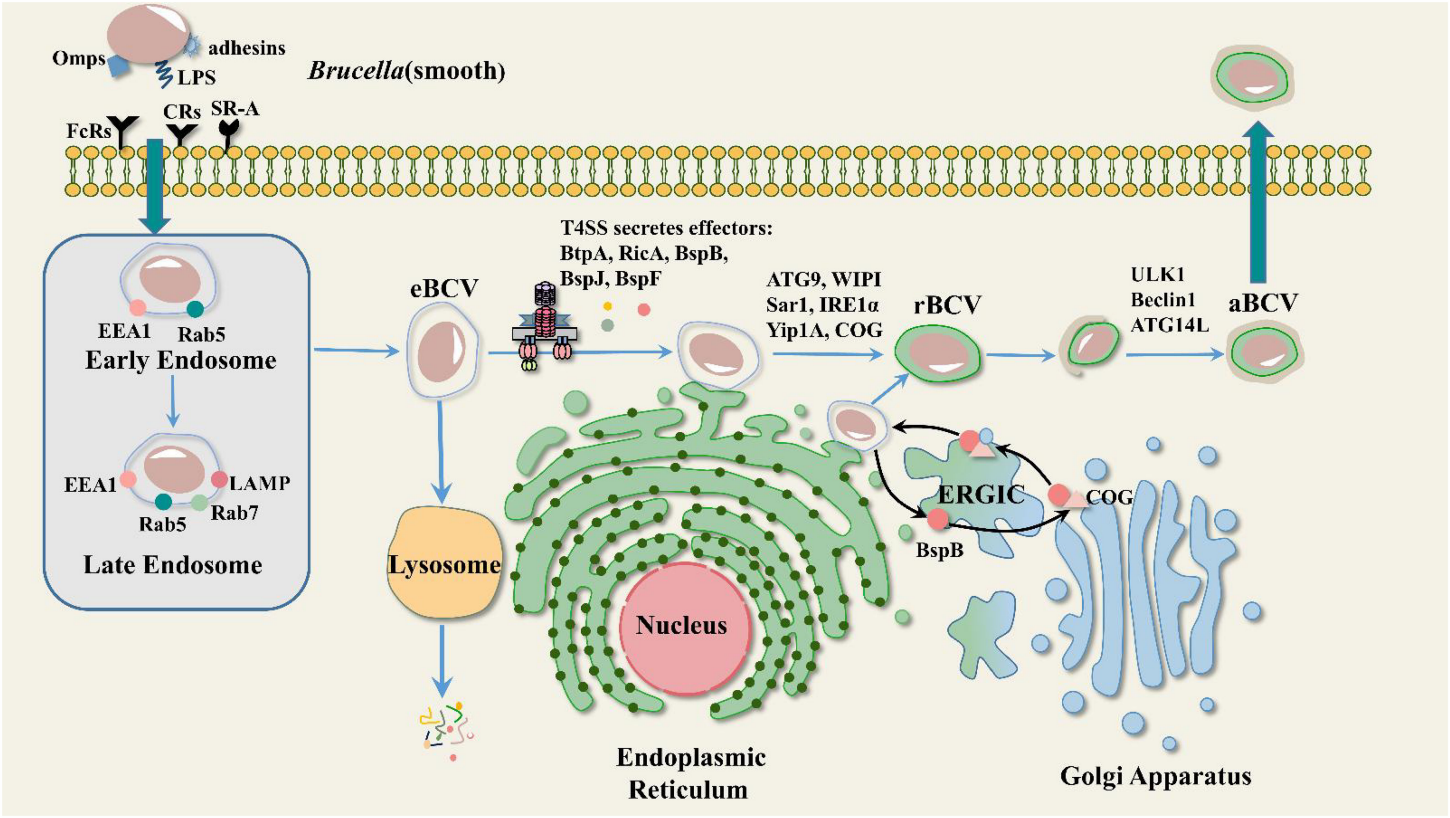
Mode of intracellular transport of *Brucella*. Heat shock protein 60 (HSP60) and LPS from Smooth *Brucella* bind to prion protein (PrPc) and class A scavenger receptor (SR-A) on lipid rafts, respectively, and thus enter the cell, where they pass through three phases: eBCV, rBCV, and aBCV. EEA1, LAMP, Rab5, and Rab7 are involved in eBCV maturation. ATG9, WIPI, Sar1, IRE1α, Yip1A, and COG are involved in rBCV formation. The T4SS effector BspB is transported to the Golgi via the ER-to-Golgi intermediate compartment (ERGIC), where it binds to the conserved oligomeric Golgi (COG) complex and facilitates the transport of Golgi membrane-derived vesicles to the BCV. ULK1, Beclin1, and ATG14L are involved in aBCV formation.

*Brucella* is capable of infecting both professional and non-professional phagocytes. Upon entering the host cell, *Brucella* is “trapped” in a compartment, forming a membrane-enclosed structure called a *Brucella*-containing vacuole (BCV). Most BCVs fuse with lysosomes and 90% of internal *Brucella* are hydrolyzed and killed. However, it is not clear how the remaining 10% escapes killing by the host. At 10-30 minutes post-infection, BCV acquires early endosomal markers such as EEA-1 (early endosomal antigen 1) and the small GTPase Rab5 (Pizarro-Cerdá et al., 1998; Chaves-Olarte et al., 2002). Early endosomal markers present on BCVs are gradually replaced by late endosomal markers (the lysosome-associated membrane protein LAMP1 and the small GTPase Rab7) at 4 hours post-infection (Celli et al., 2003; Starr et al., 2008). Over time, the endosomes mature and acidify (pH reaches 4 to 4.5), at which point the BCVs are referred to as endosomal-containing *Brucella* vacuoles (eBCVs). BCV acidification is required for *Brucella* survival and replication (Porte et al., 1999). eBCV has been shown to interact with COPII-coated structures at functional endoplasmic reticulum exit sites (ERES) (Celli et al., 2005). At 8-12 hours post-infection, eBCV gradually loses endosomal markers and acquires ER-derived membranes. Meanwhile, eBCV acquired ER membrane markers such as calcium-binding protein and Sec61β. These vacuoles have structural and functional properties of the ER that provide conditions for the growth and replication of *Brucella*, at which point the BCVs are referred to as replicative *Brucella*-containing vacuoles (rBCVs). *Brucella* undergoes extensive replication in rBCV, which is eventually captured in autophagosome-like structures and becomes autophagic BCV (aBCV). At this point, aBCV stops maturing and kills cells (**Figure 1**) (Jiao et al., 2021). We now have a preliminary understanding of the intracellular cycle of *Brucell*. However, the underlying mechanisms of each stage of *Brucella* development within the cell have not been fully explained.

### 2.2 Virulence factor of *Brucella*: T4SS

T4SS is an important virulence factor for *Brucella*, which plays an important role in the intracellular replication of *Brucella* (de Jong and Tsolis, 2012). T4SS is encoded by the *virB* operon, and the functional *virB* system is present in all *Brucella* species and remains highly conserved (O’Callaghan et al., 1999). The *virB* system consists of *virB1*-*virB12* proteins, which can be divided into four groups according to their functions: the ATPases (*virB4* and *virB11*), the core components (*virB6*-*virB10*), the surface-exposed components (*virB2* and *virB5*), and the other components (*virB1* and *virB12*) (Xiong et al., 2021). Numerous studies have shown that *virB* is essential for persistent *Brucella* infection in various host cells (Hong et al., 2000; Comerci et al., 2001; den Hartigh et al., 2004). Salcedo, S.P. et al. investigated the ability of different *Brucella* strains to infect the human trophoblast and, as expected, the replication of *B. melitensis* 16M in JEG cells was completely dependent on *virB*. Surprisingly, the *B. abortus virB* mutant was able to replicate at a low level in JEG cells (Salcedo et al., 2013), suggesting that this replication of *B. abortus* has an incomplete dependence on the *virB* type IV secretion system, and the exact mechanism that generates this incomplete dependence is not clear and requires further validation. *B. abortus* A19 was more adaptable to harsh environments (strong acidic and high-salt) in the presence of *virB* (Deng et al., 2022), this experimental result explains that *virB* facilitates the invasion of *Brucella* spp. into host cells, adapts to the intracellular complexity, and enhances bacterial survival. In addition, the *virB* mutant strain enhanced autophagy, up-regulated IL-6, and down-regulated IL-10 expression in macrophages compared to the *B. abortus* A19 parental strain.

*virB* T4SS can secrete effectors such as RicA, VceA, VecC, BtpA, BtpB, BspJ, BspG, BspA, BspB, BspC, BspE, BspF, BPE005, BPE123, BPE043, BPE275, and SepA, which can enter the host cells to function. Based on previous studies, the role of T4SS effectors can be divided into two aspects: One is affecting the formation of rBCV and the other is affecting the virulence of *Brucella* (Xiong et al., 2021). RicA was the first effector reported to interact with the small GTPase Rab2. RicA-deficient mutants showed no significant change in virulence among infected mice or HeLa cells (Ke et al., 2015), suggesting that RicA may not play a major role in the intracellular parasitism of *Brucella*. However, in HeLa cells, the *B. abortus* RicA mutant loses the late endosomal marker LAMP1 earlier than the wild-type strain, which facilitates the RicA mutant to reach the ER faster and establish a replicative ecological niche (de Barsy et al., 2011). Rab2 is important for rBCV biogenesis and bacterial replication. However, RicA may have a negative impact on Rab2 function, so that *B. abortus* RicA mutants proliferate faster in host cells. Meanwhile, it has been found that effector BspB attenuates the negative regulation of Rab2 function by RicA (Smith et al., 2020). Mutants deficient in SepA invaded macrophages more efficiently but significantly reduced *Brucella* replication capacity at an early stage, suggesting that SepA may play an integral role in the early stages of *Brucella* infection (Döhmer et al., 2014). SepA-deficient strains had a defect in excluding LAMP1, suggesting that SepA may have a role in inhibiting the fusion of BCVs with lysosomes (Ke et al., 2015). It is not clear why *Brucella* deficient in SepA invades macrophages more efficiently. Quorum sensing (QS) is a regulatory system that allows microorganisms to sense changes in population density for gene expression regulation. VjbR and BlxR are two regulators of QS. It was found that VjbR mutants down-regulated the expression of *virB* operon and flagellar genes, suggesting that VjbR contributes to *B. melitensis* survival by regulating *Brucella* virulence factors (Delrue et al., 2005). In one study (Brambila-Tapia and Pérez-Rueda, 2014), VjbR activated genes associated with persistent infection (e.g., intracellular trafficking, vesicular transport) and defense mechanisms; BlxR inhibits the expression of genes related to metabolism (e.g., energy production and conversion), which facilitates bacterial adaptation to the intracellular environment. GntR10 is a transcriptional regulator of *Brucella*. Deletion of GntR10 resulted in down-regulation of VjbR and BlxR expression, affecting the expression of T4SS effectors (BspE and BspF) and ultimately inhibiting NF-κB activation (Li et al., 2023).

## 3 *Brucella* mediates autophagy

### 3.1 Autophagy is involved in the intracellular survival of *Brucella*

There have been a number of reports suggesting that the reproduction of *Brucella* spp. in the host is inextricably linked to autophagy. Guo, F., and colleagues hold the view that autophagy benefits the intracellular survival of *Brucella* (Guo et al., 2012). In *B. melitensis* 16M-infected mouse macrophages, they observed a rise in the expression of the autophagy marker LC3-II and the formation of autophagosomes. Moreover, *Brucella* replication was significantly reduced after treatment with the autophagy inhibitor 3-methyladenine. A similar phenomenon occurred in mouse RAW264.7 macrophages infected by *B. suis* (Dong et al., 2023) i.e., *B. suis* invasion up-regulated LC3-II expression, and additionally, the autophagy-lysosomal pathway positively promoted *Brucella* proliferation. These researchers point out that autophagy may also serve bacteria as a form of self-protection. However, Hamer, I.’s experiments (Hamer et al., 2014) showed that *B. abortus* and *B. melitensis* do not induce macroautophagy in mouse embryonic fibroblasts to reach their replicative niche or to stimulate their replication. Differences in cell types may be one reason for the variability.

### 3.2 VceA and BtpB inhibit autophagy

Autophagy is an innate immune mechanism of the host cell, and *Brucella* effectors may disrupt host cell homeostasis by inhibiting autophagy. VceA, one of the first substrates identified in *Brucella* T4SS, is regulated by VjbR and remains highly conserved among all sequenced *Brucella* genes (de Jong et al., 2008). In preliminary studies, it was observed that VceA mutant strains enhanced autophagy, but the role played by VceA in the autophagy process needs to be further investigated. Zhang, J.’s team (Zhang et al., 2019) found that infection of human trophoblast cells by *B. abortus* VceA mutant strains resulted in increased expression of ATG5 and LC3-II, and decreased levels of P62 and LC3-I expression. They observed the expression of P62 protein under immunofluorescence microscopy and found that the number of P62 protein focal points was significantly reduced in the mutant strain group. In addition, the number of autophagosomes was increased in the VceA knockout group under electron microscopy. It is therefore hypothesized that VceA may have a role in inhibiting autophagy. Li, J et al. found that deletion of BtpB resulted in increased LC3-II expression, decreased P62, and accumulation of autophagic lysosomes (Li et al., 2022). This suggests that BtpB also has an inhibitory effect on autophagy. *Brucella* Omp25 affects macrophage autophagy (Jiao et al., 2020), but the molecular mechanism is unknown.

### 3.3 Autophagy proteins (WIPI1, ATG9, ULK1, Beclin1) are involved in *Brucella* intracellular replication

Autophagy proteins play an important role in autophagy genesis (**Table 1**). Taguchi, Y.’s experiments confirmed that cells knocking down the expression of the autophagosome nucleation protein WIPI1 and the autophagy protein ATG9 had significantly lower ER membrane-derived large vacuole production (Taguchi et al., 2015). This suggests that WIPI1 and ATG9 are involved in rBCV biogenesis. In addition to this, the autophagy proteins ULK1 and Beclin1 are involved in *Brucella* intracellular parasitism. ULK1 is a serine/threonine protein kinase that plays a key role in the initiation of autophagy and serves as a homologue of yeast Atg1 in mammals. Five ULK1 homologs have been identified, and they are ULK1, ULK2, ULK3, ULK4, and STK36, of which only ULK1 and ULK2 are widely believed to be involved in the regulation of the conventional autophagy signaling pathway (Zachari and Ganley, 2017). In the absence of serum, neuronal autophagy was induced by simulating an environment with low potassium concentrations, and the results showed that ULK1 was required, whereas ULK2 was not (Lee and Tournier, 2011). There are also several experiments confirming that ULK1 is indispensable and that the absence of ULK1 is sufficient to disrupt the onset of conventional autophagy (Chan et al., 2007), whereas ULK2 acts as a surrogate or compensatory for the impairment of ULK1 function in this process (Zachari and Ganley, 2017). ULK1 and ULK2 interact with the same core components and share a high degree of similarity in their protein kinase domains, i.e. 52% protein sequence and 78% homology. It is unclear why ULK1 dominates the autophagy pathway. In yeast, the Atg1 complex consists of Atg1, Atg11, Atg13, Atg17, Atg29, Atg31. In mammals, the components of the ULK1 complex are ULK1, ATG13, ATG101, FIP200, (200 kDa focal adhesion kinase family interacting protein)/RB1CC1. Interaction of ATG13 and FIP200/RB1CC1 with ULK1 helps enhance the activity and stability of ULK1 kinase (Ganley et al., 2009). During conventional autophagy, when the organism is in an abnormal (e.g., starvation) state, mTORC1 activity is inhibited and AMPK activates ULK1 activity by phosphorylating (Kim et al., 2011). Beclin1 is one of the important components of class III phosphatidylinositol 3-kinase (PI3P), and although it is an important regulator of autophagy initiation, its specific mechanism of action remains poorly studied. Pandey, A. et al. found that in autophagy-deficient cells (including deletion or inactivation of ULK1, Beclin1, and ATG9a), intracellular transport and replication of *B. melitensis* 16M is severely disrupted (Pandey et al., 2018). However, in *B. abortus*-invaded HeLa cells (Starr et al., 2012), depletion of ULK1 and Beclin1 did not affect *Brucella abortus* delivery to the ER and bacterial replication but significantly reduced aBCV production. The two studies yielded markedly disparate findings with regard to the impact of ULK1 and Beclin1 deletions on rBCV biogenesis and *Brucella* replication. Differential behavior of *Brucella* in macrophages and non-phagocytes (e.g., HeLa) has been demonstrated. Thus, differences in cell models may be one of the possibilities for producing different results. Nevertheless, the results of both Pandey, A. and Starr point out that ULK1 and Beclin1 have an effect on *Brucella* parasitism. However, the effect of ULK1- and Beclin1-mediated autophagy mechanisms on *Brucella* still needs to be further studied.

**Table 1:** Core autophagy proteins in autophagosome formation.

	Yeasts	Mammals	Characterization of Autophagy Proteins in Mammals	Effects in *Brucella*-infected HeLa Cells
Atg1/ULK1 complex	Atg1 Atg11 Atg13 Atg17 Atg29 Atg33	ULK1 ATG13 FIP200, RB1CC1 C12or144, ATG101	The ULK1 complex is regulated by mTOR and AMPK, and AMPK induces autophagy by directly phosphorylating ULK1 (Kim et al., 2011). ATG13 binds to ULK1 and FIP200 through the C-terminal domain (CTD), and the CTD of ULK1 binds to FIP200 (So Many Roads: the Multifaceted Regulation of Autophagy Induction, 2024). Interaction of ATG13 and FIP200 with ULK1 contributes to increased ULK1 kinase activity and stability, and is essential for proper localization of ULK1 to autophagosomes (Ganley et al., 2009). ATG101 can interact with ATG13 and FIP200. The formed ULK1 complex has a role in recruiting subsequent autophagy proteins and separating membranes for nucleation (Tooze et al., 2010).	ULK1 is required for aBCV formation and is dispensable for rBCV formation (Starr et al., 2012).
Atg9 and its circulatory system	Atg2	ATG2	ATG2 is essential for autophagy, a lipid transfer protein that functions at the ER-autophagosome interface, with roles in closing autophagosome-related membranes and regulating lipid droplet size and distribution (Velikkakath et al., 2012; Valverde et al., 2019).	
	Atg9	ATG9	A transmembrane protein and lipid scrambase that mediates autophagosomal membrane expansion; circulating transport between the Golgi and endosomes via vesicles (Webber et al., 2007; Matoba et al., 2020).	Required for rBCV biogenesis (Taguchi et al., 2015).
	Atg18	WIPI1/2	PtdIns3P effector, which recruits the ATG12-ATG5-ATG16L complex, mediates LC3 lipidation (Polson et al., 2010; Proikas-Cezanne et al., 2015).	Required for rBCV biogenesis (Taguchi et al., 2015).
PtdIns3K complex	VPS34	PIK3C3, VPS34	Beclin1 homodimerization tends to bind to Bcl-2 and inhibit autophagy, its monomer binds to VPS34, VPS15, Atg14, and NRBF2 to regulate autophagy, and complexes formed with VPS34, VPS15, UVRAG, and Bif1 are involved in endocytosis trafficking (Levine et al., 2015).	
	VPS15	PIK3R4, VPS15		
	VPS30, Atg6	Beclin1		Beclin1 is required for aBCV formation and is dispensable for rBCV formation (Starr et al., 2012).
	Atg14	ATG14		ATG14L is required for aBCV formation (Starr et al., 2012).
Atg8 conjugate system	Atg8	LC3A/B/C, GABARAP, GATE-16	Such proteins are cleaved by the hAtg4B protease to expose Gly residues that bind to PE (Tanida et al., 2004). LC3 is involved in phagophore membrane elongation, while GABARAP and GATE-16 are involved in autophagosome maturation (Weidberg et al., 2010).	LC3B is dispensable for rBCV and aBCV formation (Starr et al., 2012)
	Atg7	ATG7	E1-like enzymes, ATG7-deficient cells are still able to form autophagosomes, i.e., the ATG7-independent alternative pathway (Nishida et al., 2009).	ATG7 is dispensable for rBCV and aBCV formation (Starr et al., 2012).
	Atg3	ATG3	E2-like enzyme	
	Atg4	ATG4A/B/C/D	Cysteine protease, delipidating enzyme (Tanida et al., 2004).	ATG4B is dispensable for aBCV formation (Starr et al., 2012).
Atg12 conjugate system	Atg12	ATG12	The ATG12-ATG5-ATG16L complex recruits ATG3-LC3 family proteins to the membrane, mediates the lipidation reaction and promotes their binding to PE (Lystad et al., 2019).	
	Atg5	ATG5		ATG5 is dispensable for rBCV and aBCV formation (Starr et al., 2012)
	Atg16	ATG16L1/2		ATG16L1 is dispensable for aBCV formation (Starr et al., 2012)
	Atg7	ATG7	E1-like enzyme	
	Atg10	ATG10	E2-like enzyme	

## 4 Inflammatory mechanisms of host invasion by *Brucella*

### 4.1 VceC induces inflammatory responses

**Figure 2:**
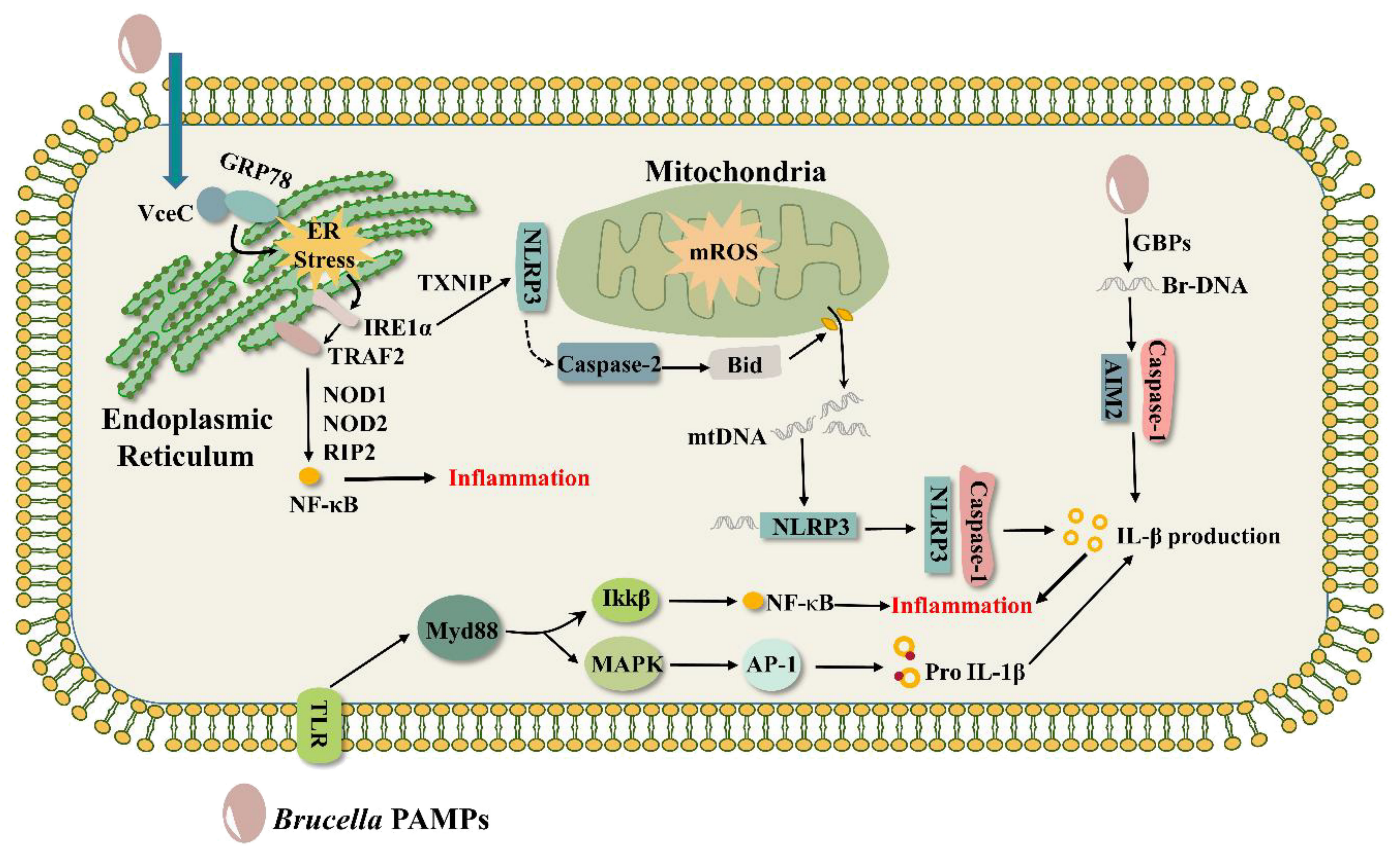
*Brucella* triggers inflammatory responses. VceC binds to the ER chaperone immunoglobulin GRP78 to trigger ER stress, activates the IRE1α, translocates TRAF2 to the ER membrane, and activates the NF-κB pathway with the support of the signaling molecules NOD1, NOD2, and the adaptor protein RIP2, inducing an inflammatory response. AIM2 recognizes *Brucella* DNA in the presence of the guanylate-binding protein GBP and activates the inflammatory response of the caspase-1 pathway. During ER stress, IRE1α is activated to recruit TXNIP and NLRP3 in mitochondria to participate in mitochondrial ROS. At the same time, NLRP3 activates Caspase-2 to make the BH3 protein Bid a truncated active form, induces the release of DNA from mitochondria, and DNA interacts with NLRP3 to activate the inflammatory response of the Caspase-1 pathway. Other pathogen-associated molecular patterns (PAMPs) of *Brucella*, such as LPS, major outer membrane proteins, and lipoproteins, trigger inflammatory responses via the TLR pathway.

Like VceA, VceC is conserved in all sequenced *Brucella* genes, and its C-terminal 20 amino acids are essential for the translocation of VceC into the host cell (de Jong et al., 2008). VceC translocates to the ER through its structural advantage and binds to the ER chaperone immunoglobulin GRP78 to cause ER stress, inducing an unfolded protein response (UPR) as well as ultimately leading to an inflammatory response (de Jong et al., 2013; Keestra-Gounder et al., 2016). The UPR has three signaling pathways involving three important ER transmembrane proteins: inositol-requiring enzyme 1α (IRE1α), protein kinase RNA-like endoplasmic reticulum kinase (PEPK), and activating transcription factor 6 (ATF6) (Schröder and Kaufman, 2005). VceC-triggered inflammatory responses are mainly realized through the IRE1α pathway (Keestra-Gounder et al., 2016). The IRE1α pathway is activated in a time-dependent manner during *Brucella* infection, and activated IRE1α translocates TRAF2 to the ER membrane, which subsequently sends signals to activate the NF-κB pathway, eventually inducing an inflammatory response (**Figure 2**) (Taguchi et al., 2015; Caruso and Núñez, 2016; Keestra-Gounder et al., 2016). Notably, the process of NF-κB activation requires the support of NOD1 and NOD2 signaling molecules with the activity of the adaptor protein RIP2. NOD1 and NOD2 are two members of the NOD-like receptors (NLRs) family of pattern recognition receptors (PRRs) that sense bacterial peptidoglycan (PGN)-derived fragments to induce pro-inflammatory responses (Caruso and Núñez, 2016). In contrast, NOD1 and NOD2 were shown to be irrelevant to PGN stimulation during VceC-induced inflammation. Although NOD1 and NOD2 play an important role in ER stress-induced inflammatory responses, it has been found that these receptors do not affect *Brucella* parasitism in mice (Oliveira et al., 2012). *B. abortus* infected normal (C57BL/6), NOD1-deficient, NOD2-deficient, and RIP2-deficient mice, respectively, and there was no difference in the bacterial numbers in the spleens of the four groups, suggesting that NOD1, NOD2, and RIP2 are dispensable for the intracellular survival of *B.abortus*. The second is that NOD1 and NOD2 are not the root cause of host cell (trophoblast) death. Infection of pregnant mice lacking NOD1 and NOD2 with *B. abortus* did cause suppression of the inflammatory response and increased pup survival compared to pregnant wild-type mice (Keestra-Gounder et al., 2016). However, the TUNEL assay of placental sections from *B. abortus*-infected NOD1- and NOD2-deficient mice as well as control mice showed no significant difference in the number of trophoblast cell deaths between the two groups, indicating that NOD1 and NOD2 do not play a role in trophoblast cell death (Byndloss et al., 2019).

### 4.2 BtpA and BtpB regulate the inflammatory response

BtpA and BtpB are known to inhibit the host’s innate immune response and regulate the inflammatory response. BtpA, which is not present in *B. suis*, contains a TIR domain at its C-terminus, and the TIR domain is an important component of toll-like receptor (TLR)-mediated innate immunity. BtpA is similar to the TIR domain adapter protein MAL and can compete with MAL for TLR4. Meanwhile, BtpA interacts with MAL to reduce MAL phosphorylation and eventually reduces TLR4 and TLR2-mediated activation of the NF-κB pathway (Radhakrishnan et al., 2009; Sengupta et al., 2010; Alaidarous et al., 2014). In addition, BtpA can interact with the TIR domain-containing adapter protein MyD88 to influence the inflammatory response (Chaudhary et al., 2012). BtpA attenuates LPS-induced pyroptosis and inflammatory cytokine secretion by ubiquitination and degradation of caspase-1, 4, 11 in mouse and human macrophages (Jakka et al., 2017). BtpA inhibits caspase- 4/11-mediated inflammation, which is the far-reaching inspiration for the development of therapeutic drugs targeting LPS-induced septicemia. BtpB is present in all sequenced *Brucella* species and, similar to BtpA, also contains a TIR domain that can function accordingly by interfering with TLR signaling. In addition, *Brucella* flagella can evade TLR5 recognition to limit innate immune recognition (Terwagne et al., 2013).

### 4.3 Inflammatory responses triggered by NLRP3 and AIM2

It has been shown that ASC inflammasomes, mainly NLRP3 and AIM2, can sense *Brucella* and regulate caspase 1-mediated inflammation (**Figure 2**) (Gomes et al., 2013), ultimately influencing *Brucella* pathogenesis. Generally, the C-terminus of AIM2 senses pathogen DNA, and its N-terminal pyrin domain binds to the N-terminus of ASC, while the C-terminal CARD domain of ASC binds to the procaspase-1. Formation of the DNA-AIM2-ASC-pro-Caspase-1 complex, the AIM2 inflammasome, regulates IL-1β maturation and secretion through activation of caspase-1 (Hornung et al., 2009). It was found that AIM2 recognizes *Brucella* DNA (Costa Franco et al., 2019) and that guanylate-binding protein (GBP) plays an important role in this process (Costa Franco et al., 2018), possibly by killing *Brucella* so that the DNA is released in large quantities for AIM2 binding. NLRP3 crosstalks with the ER stress IRE1α pathway for cytokine IL-1β secretion and pyroptosis (Bronner et al., 2015). T4SS effectors (VceC, BtpA, BspL), Yip1A (a host factor), and STING activate the IRE1α axis of the UPR (de Jong et al., 2013; Smith et al., 2013; Taguchi et al., 2015; Guimarães et al., 2019; Luizet et al., 2021), but it is not clear whether they can act directly on NLRP3-triggered inflammatory responses. During *Brucella* infection, IRE1α is activated to recruit TXNIP and NLRP3 in mitochondria to engage in mitochondrial ROS. Simultaneously, NLRP3 activates Caspase-2 to process the BH3-only protein BID into its truncated active form, which subsequently induces the creation of a pore in the mitochondrial membrane and the release of mitochondrial DNA. DNA binds to NLRP3 and promotes the formation of the NLRP3-ASC-Caspase-1 complex, eventually leading to IL-1β production (Shin and Argon, 2015; Marim et al., 2017). Mice with AIM2, NLRP3, and ASC deletions were more susceptible to *B. abortus* compared to wild-type strain (Gomes et al., 2013; Tupik et al., 2020), suggesting that multiple ASC-dependent inflammasomes attenuate *Brucella* pathogenesis and contribute to host protection from infection. Surprisingly, AIM2 and NLRP3 were dispensable for controlling *Brucella* joint burdens, but Caspase-1 and Caspase-11 caused arthritis and apoptosis to control *Brucella* joint infection. In addition to this, LPS can also induce caspase-11-mediated pyroptosis (Lacey et al., 2018).

### 4.4 cGAS-STING pathway induces type I interferon production

The cyclic GMP-AMP synthase (cGAS)-stimulator of interferon genes (STING) signaling pathway is strongly associated with various inflammatory diseases (Decout et al., 2021). STING is an important DNA sensor that does not recognize whole DNA but can directly recognize cyclic dinucleotides (CDNs) of bacterial origin. So, STING usually works through two different mechanisms. On the one hand, STING directly recognizes CDN (e.g., c-di-GMP) and induces an intracellular immune response. On the other hand, DNA from pathogenic microorganisms is released into the host cytoplasm (Burdette et al., 2011). cGAS recognizes and binds to the DNA, catalyzes the production of cGAMP, activates STING, and ultimately induces the expression of genes such as type I interferons and pro-inflammatory cytokines (Ishikawa et al., 2009; Sun et al., 2013). In recent years, researchers have found that STING plays an important role in controlling *Brucella* infection *in vitro* and *in vivo*, while cGAS is dispensable (Costa Franco et al., 2018; Alonso Paiva IM et al., 2023). Costa Franco et al. found that *Brucella* DNA is not only dependent on STING to induce expression of innate immunity genes (IFN-β and GBP), but also induces STING translocation from the ER to the perinuclear region. In addition, they observed that IRF3 and the P65 subunit of NF-κB were phosphorylated and translocated into the nucleus, a process that is also STING-dependent. These results suggest that *Brucella* invasion of host cells also induces the STING pathway. This study also demonstrates that *Brucella* produces the second messenger c-di-GMP to activate the STING pathway directly. They propose that *Brucella* first produces c-di-GMP, which induces initial STING signaling and activation of type I IFN and GBP. GBP binds to BCV, BCV lyses and releases *Brucella* DNA. cGAS recognizes the DNA and produces cGAMP, which further amplifies the STING signal. It is undeniable that STING plays an important role in defense against *Brucella* infection, but the mechanism of the cGAS-STING axis in *Brucella* infection needs to be further validated, and how GBP and BCV binding induces BCV lysis is unclear. The researchers found that Omp25 is dependent on ubiquitin-proteasome degradation of cGAS, inhibits activation of the cGAS/STING signaling pathway, and interferes with the production of IFN-β, thereby evading host innate immunity mechanisms (Li et al., 2021).

### 4.5 Lipopolysaccharide regulates inflammation and controls host immunity

LPS is closely related to inflammation. *B. melitensis*, *B. abortus*, as well as *B. suis* express smooth LPS (S-LPS), which consists of lipid A, core oligosaccharide, and polysaccharide O-chain. *B. ovis* and *B. canis* become naturally rough LPS (R-LPS) due to lack of O-chain. The presence of the O-chain protects *Brucella* from damage by host cell cationic peptides, oxygen metabolites, and complement (Cardoso et al., 2006). The LPS O-chain interacts with the lipid raft on the host cell surface, allowing *Brucella* to enter the cell and delaying fusion with lysosomes to promote *Brucella* survival (Porte et al., 2003). O-chain also inhibits phagocytic apoptosis (Fernandez-Prada et al., 2003), which favors bacterial survival by avoiding host cell apoptosis. Unlike *Enterobacteriaceae*, the lipid A portion of *Brucella* and *Ochrobactrum anthropi* is recognized by TLR4 only at high concentrations, eliciting biochemical signals, whereas TLR-1, TLR-2, and TLR-6, as well as their heterodimeric combinations, are not (Dueñas et al., 2004). Compared to rough strains, lipid A of smooth *Brucella* was more able to trigger TLR4 in CHO cells and was more advantageous in inducing dendritic cell maturation (Campos et al., 2004). *Brucella* lipid A has ultra-long-chain fatty acids (ULCFAs) and is structurally different from typical lipid A. The typical lipid A activates innate immune defenses by binding to TLR4 and the TLR4 25-kDa co-receptor MD-2 (Meng et al., 2010). ULCFAs may result in *Brucella*’s ability to bind to TLR4 being greatly attenuated and failing to stimulate a strong inflammatory response (Lapaque et al., 2006), which serves as a way for *Brucella* to conceal itself and escape killing (Conde-Álvarez et al., 2012). The lipid A portion of *B. abortus* induces the death of polymorphonuclear neutrophils (PMNs), and the dead PMNs are removed by phagocytosis. At the same time, *Brucella* carried by PMNs may be transferred to other organs for the next round of proliferation (Barquero-Calvo et al., 2015). This manner of PMN death not only facilitates a safe pathway for *Brucella* to spread in host cells but also hinders the pro-inflammatory signaling pathway. LPS core connects O polysaccharide and lipid A. WadA, WadB, WadC, and WadD are *Brucella* LPS core glycosyltransferases involved in assembling the LPS core branching structure (Shi et al., 2022). Moreover, WadC gene mutation caused increased binding of TLR4 to MD-2 (Conde-Álvarez et al., 2012; Gil-Ramírez et al., 2014; Soler-Lloréns et al., 2014; Salvador-Bescós et al., 2018). In conclusion, the LPS core helps *Brucella* to evade the host immune system and favors its survival.

### 4.6 Physiologic functions of outer membrane proteins

Lipoproteins are key mediators of the pro-inflammatory response induced by *Brucella* (Giambartolomei et al., 2004)*. Brucella* strains produce three outer membrane lipoproteins: Omp10, Omp16, and Omp19. Omp16 has a peptidoglycan-associated lipoprotein (Pal) domain that is highly conserved in *Brucella*. Inactivation of Omp16 damages the integrity and activity of the *Brucella* outer membrane, resulting in decreased intracellular survival of *Brucella* in macrophages (Zhi et al., 2020). Some researchers have noted that while Omp10 and Omp19 are not essential genes for *Brucella* intracellular survival *in vitro*, Omp10 mutants lead to a significant attenuation of *Brucella* survival in mice and Omp19 inactivation alters *Brucella* outer membrane properties (Tibor et al., 2002). After *B. abortus* infects the host via the oral cavity, Omp19 protects the bacteria from gastrointestinal proteases and lysosomal proteases. Not only that, Omp19 has a protective role for the outer membrane protein Omp25, preventing Omp25 from being degraded by proteases (Pasquevich et al., 2019). Lipid-modified Omp16 and Omp19 in *B. abortus* induced macrophages to produce TNF-α, IL-6, IL-10, and IL-12, with the involvement of TLR2 (Giambartolomei et al., 2004). In conclusion, Omp10, Omp16, and Omp19 are essential for the survival of *Brucella*.

### 4.7 Omp25 inhibits the production of pro-inflammatory factors

Omp25 is an outer membrane protein of *Brucella*. Omp25 can manipulate innate immunity to establish chronic infections (Degos et al., 2020). *In vitro*, Omp25 of *B. abortus* binds directly to the SLAMF1 receptor on the surface of dendritic cells (DCs), restricting NF-κB nuclear translocation and inhibiting the secretion of pro-inflammatory factors, although this does not affect intracellular replication and transport of *Brucella abortus*. *In vivo*, Omp25 binding to SLAMF1 does not affect *Brucella* replication during the acute infection phase but favors bacterial persistent survival. The researchers also found that Omp25 inhibited LPS-induced IL-12 production in human monocytes (Cui et al., 2017). Omp25 inhibition of TNF-α was associated with Omp25-induced miRNAs in *B. suis*-infected porcine and mouse macrophages (Luo et al., 2017). On the one hand, the relevant miRNAs acted on the 3’UTR region of TNF-α to inhibit TNF-α production at the transcriptional level; on the other hand, the miRNAs targeted IRAK1 and TRAF6 proteins to prevent the NF-κB signaling pathway from being activated, thus reducing TNF-α production, which ultimately favored *Brucella* survival.

## 5 *Brucella* regulates apoptosis

### 5.1 *Brucella* infection promotes or inhibits apoptosis

*Brucella* infection promotes apoptosis (**Table 2**). *B. Melitensis* 16M infection induces apoptosis via ROS (Li et al., 2016). Reactive oxygen species (ROS) are the second messengers of apoptosis. When cells receive pro-apoptotic signals, ROS production increases, calcium influx increases, Bax up-regulates, and the mitochondrial permeability transition pore (MPTP) opens, activating the trypsin, which leads to cell death (Sun et al., 2016). It has been shown that S-type *Brucella* inhibits apoptosis and R-type *Brucella* promotes apoptosis. Compared to R-type *Brucella*, O-polysaccharide (OPS) was present in the outer membrane of S-type *Brucella*. It has been proposed (Fernandez-Prada et al., 2003) that OPS prevents the death of macrophages (the preferred target of *Brucella* intracellular replication), which ultimately favors *Brucella* survival; *Brucella* deficient in OPS not only leads to the death of the bacteria itself, but also promotes phagocytic apoptosis. The complement system is a multimolecular system composed of many types of proteins, and the host can activate the complement pathway to clear most Gram-negative bacteria. It is known that the unique structure of *Brucella* OPS hinders the binding of complement factors to the cell membrane of bacteria, favoring the survival of *Brucella* (Barquero-Calvo et al., 2007). We speculate that OPS may prevent macrophage apoptosis by reducing the chances of *Brucella* being recognized by relevant host cell receptors or cytokines, just as complement factors are impeded from binding to the cell membrane of bacteria.

**Table 2:** Temporal expression of apoptosis-regulating proteins and the *Brucella’s* control of apoptosis.

Name	Regulatory processes	Conclusion
ROS	The apoptosis rate was significantly reduced in the NAC (ROS eliminator) treated group at 3, 6, 12, and 24h post-infection, and the expression of Caspase3 was decreased in the NAC treated group at 3, 6, 12, and 24h post-infection.	ROS-3h, 6h, 12h, 24h (Time of *Brucella* infection of cells)- promote apoptosis
VceA	Compared to the parental strain, ΔVceA decreased Caspase3 (pro-apoptotic) expression at 24h post-infection, increased Bcl-2 (anti-apoptotic) expression at 12h and 24h post-infection, and decreased the percentage of apoptotic cells at 24h post-infection.	VceA-24h-promote apoptosis
BtpB	ΔBtpB significantly decreased the average apoptosis rate of early apoptotic cells at 48 h post-infection. After 48h of pEGFP-C1-BtpB transfected cells, there were more TUNEL-positive cells in the BtpB-transfected group than in the pEGFP-C1 group, and the expression of Caspase3 was enhanced in the BtpB-transfected group.	BtpB-48h-promote apoptosis
VceC	ΔVceC increased the proportion of early apoptosis in GTC cells at 12h and 48h post-infection, and elevated the levels of apoptosis-associated proteins (Caspase3, Chop) at 24h and 48h post-infection.	VceC-48h-inhibit apoptosis
BspJ	ΔBspJ significantly increased the rate of apoptosis at 24 h post-infection.	BspJ-24h-inhibit apoptosis
BspG	ΔBspG significantly increased the rate of apoptosis at 24 h post-infection.	BspG-24h-inhibit apoptosis
BspF	ΔBspF significantly increased the proportion of early apoptotic cells at 24h and 48h post-infection and significantly increased the expression levels of pro-apoptotic proteins (Caspase3, AIF, Bax) at 24h and 48h post-infection.	BspF-24h, 48h-inhibit apoptosis
Omp25	Different concentrations of recombinant Omp25 protein were incubated with BV cells for 24 h. The percentage of apoptotic cells decreased as the concentration of Omp25 protein increased.	Omp25-inhibit apoptosis
Omp31	ΔOmp31 triggered more apoptosis at 2-24h post-infection. ΔOmp31 increased the expression of Caspase3, Caspase8 and Caspase9 at 4h post-infection, decreased the expression of Bcl-2 at 8, 12, and 24h post-infection, increased Bax expression at 2, 4, 8, 12, and 24 h post-infection, and increased Cyt c concentration at 4, 8, 12, and 24 h post-infection.	Omp31-4h, 8h, 12h, 24h-inhibit apoptosis
A20	At 8h post-infection, NF-κB was inhibited using PDTC, and the A20 knockdown group was able to promote apoptosis.	A20-inhibit apoptosis
Ca^2+^/Nedd4/Calpain2	Calcium ion concentration gradually increases 4-24h after *Brucella* infection of cells, Calpain2 binds to Nedd4 and is ubiquitinated at 24h post-infection.	Ca^2+^/Nedd4/Calpain2-24h-inhibit apoptosis

**Figure 3:**
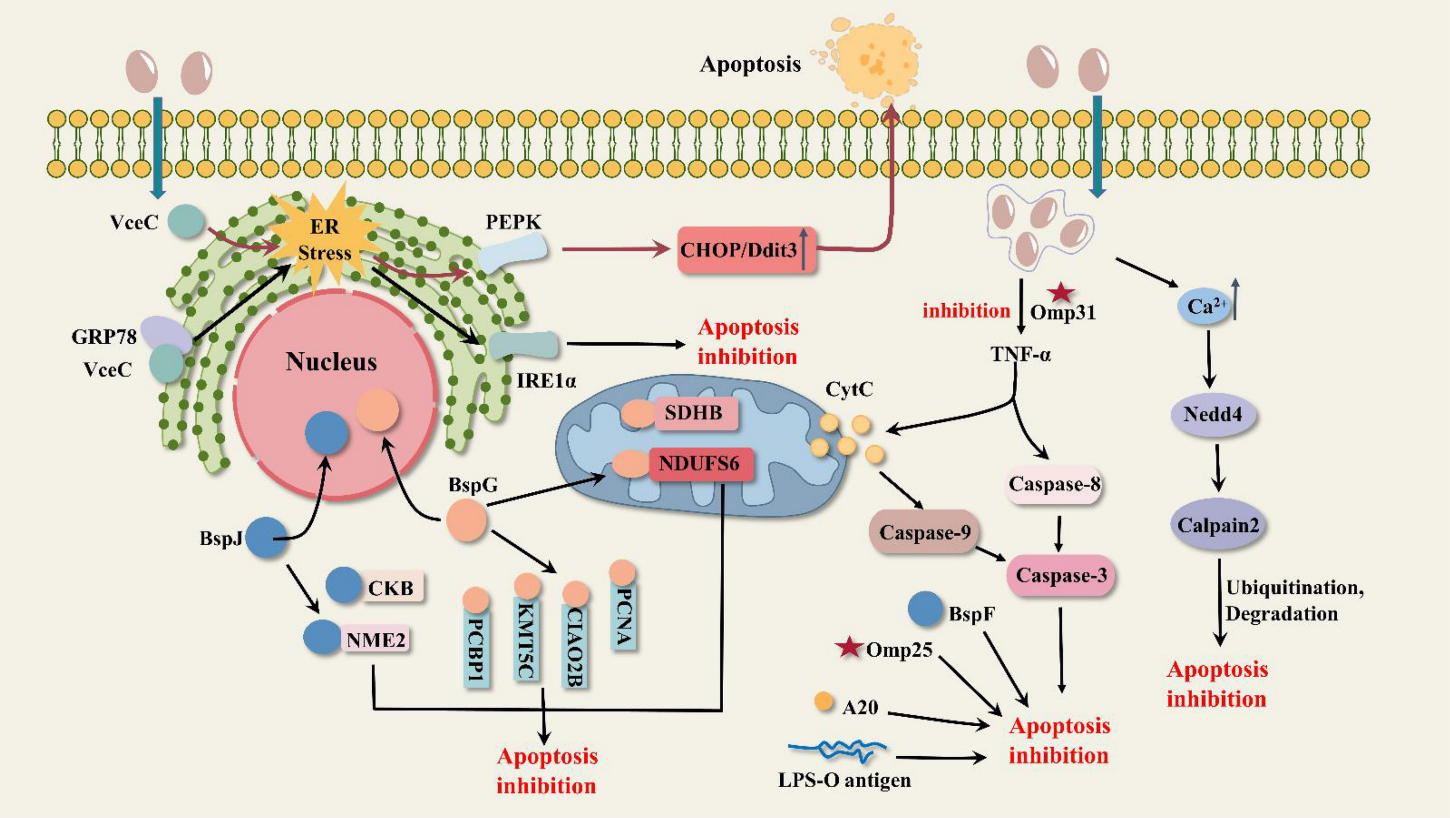
Possible pathways of apoptosis inhibition by *Brucella*. VceC triggers ER stress and activates the PEPK metabolic pathway to induce apoptosis; in addition, the activated IRE1 pathway inhibits apoptosis. BspJ interacts with host proteins NME2 and CKB to inhibit apoptosis. BspG interacts with host proteins PCBP1, KMT5C, NDUFS6, PCNA, CIAO2B, and SDHB to inhibit apoptosis. BspF inhibits apoptosis by affecting P53 expression. The outer membrane protein Omp31 inhibits the TNF-α-CytC-Caspase9 mitochondrial apoptotic pathway and the TNF-α-Caspase8-Caspase3 classical apoptotic pathway. *Brucella* infection leads to increased calcium ion concentration, activation of the E3 ubiquitin ligase Nedd4, promotion of calpain ubiquitination and degradation, and inhibition of the Caspase-3 apoptotic pathway. The outer membrane proteins Omp25 and LPS-O polysaccharide inhibit apoptosis; zinc finger protein A20 is involved in apoptosis but is not the only factor.

*Brucella* infection inhibits apoptosis (**Table 2**; **Figure 3**). Gaidiero et al. found that *B. abortus* 19 infection of monocytes and lymphocytes resulted in delayed apoptosis compared to healthy controls (Galdiero et al., 2000). Tumor necrosis factor (TNF-α) plays an important role in inducing apoptosis. Gross et al. found that *B. suis* infected human monocytes *in vitro* to inhibit apoptosis non-dependently with TNF-α (Gross et al., 2000). The results of these two studies suggest that *Brucella* invasion of host cells exhibits apoptosis inhibition, but it has not been elucidated how *Brucella* regulates anti-apoptotic mechanisms. In *B. melitensis*-infected mouse macrophages, the expression of many mitochondrion-associated genes involved in the apoptotic pathway was down-regulated (He et al., 2006). It is therefore speculated that *Brucella* inhibits macrophage apoptosis possibly related to the inhibition of mitochondrial gene expression involved in cytochrome C release, ROS production, and mitochondrial membrane permeability. Cui, G., and colleagues found that the E3 ubiquitin ligase Nedd4 is required for *B. abortus* survival in macrophages and can regulate apoptosis (Cui et al., 2014). This process can be briefly described as follows: *Brucella* infection leads to an increase in intracellular calcium ion concentration and activation of Nedd4, which promotes ubiquitination and degradation of calpain and inhibits the activation of caspase-3, ultimately inhibiting apoptosis. The development of calcium channel blockers or drugs that inhibit the increase of calcium ions may be a novel idea for the treatment of brucellosis. Zinc finger protein A20 is a dual inhibitor of macrophage activation and apoptosis, both promoting apoptosis and acting as an anti-apoptotic protein. A20 promotes the intracellular growth of *B. abortus* by inhibiting macrophage activation and apoptosis (Wei et al., 2015).

### 5.2 VceA and BtpB promote apoptosis

In experiments with *B. abortus* VceA mutant strains infecting human trophoblast cells, the researchers observed a decrease in the level of cysteine-3 (an early marker of apoptosis) and an increase in the expression of Bcl-2 (an anti-apoptotic gene) (Zhang et al., 2019). This suggests that VceA may have a role in promoting apoptosis. Study shows that BtpB triggers apoptosis. In Li, J’s experiments (Li et al., 2022), BtpB plasmid was transfected into RAW264.7 cells, observing significant DNA fragmentation and a rise in caspase-3 expression, which is a typical feature of apoptosis. Moreover, flow cytometry showed that BtpB-deficient *B. suis* inhibited macrophage apoptosis after 48h of infection, suggesting that BtpB induces apoptosis, but its cellular targets and specific mechanisms are unknown.

### 5.3 VceC promotes or inhibits apoptosis through different pathways

VceC not only induces inflammatory responses but also affects host cell apoptosis. C/EBP-homologous protein (CHOP) is the most important apoptotic pathway mediated by the UPR. VceC regulates the apoptosis pathway of CHOP and affects *Brucella* proliferation in cells. M. X. Byndloss et al. found that in *B. abortus*-infected pregnant mice, VceC promotes CHOP production by inducing ER stress to activate the PEPK pathway, which ultimately benefits host cell death and bacterial discharge (Byndloss et al., 2019). However, F. Zhi et al. indicated that VceC interaction with GRP78 triggers the IRE1α pathway of ER stress and inhibits apoptosis in trophoblast cells of *B. suis*-infected goats. Moreover, VceC promoted the sustained proliferation of *Brucella* in host cells, which may be attributed to the inhibition of apoptosis by *Brucella* (Zhi et al., 2019). Therefore, there is no consensus on how VceC regulates the CHOP apoptotic pathway, and its specific mechanism still requires extensive research.

### 5.4 BspJ, BspG, and BspF inhibit apoptosis

BspJ and BspG are nucleomodulins of *Brucella*. BspJ is the first protein defined that enters the nucleus of the host cell after being secreted by *B. abortus*. BspJ interacts with nucleoside diphosphate kinase 2 (NME2) and creatine kinase B (CKB). NME2 and CKB are associated with energy synthesis and have inhibitory effects on apoptosis. In a *B. abortus* 2308 infected macrophage model (Ma et al., 2020), loss of BspJ significantly increased macrophage apoptotic rate and reduced intracellular survival of *Brucella*. Suggesting that BspJ may act as a potential virulence factor to protect the intracellular survival of *Brucella* and has a role in inhibiting apoptosis in host cells. This inhibitory effect is probably achieved by interacting with NME2 and CKB. To further validate the function of BspJ in *Brucella* intracellular infection, Ma, Z.’s team (Ma et al., 2022b), did a more detailed study, and they found that deletion of BspJ reduced the survival and proliferation of *B. abortus* in the rBCV phase. Moreover, BspJ does not affect bacterial invasion and adhesion. Additionally, loss of BspJ resulted in abnormal secretion of inflammatory factors (IL-6, IL-1β, IL-10, IFN-γ, TNF-α) in host cells and mice compared to normal strains. However, it remains difficult to explain how BspJ leads to a reduction in *Brucella* survival and the mechanisms by which BspJ affects the level of apoptosis in host cells still require considerable research. Like BspJ, BspG inhibits apoptosis and interacts with host proteins PCBP1, KMT5C, NDUFS6, PCNA, CIAO2B, and SDHB. NDUFS6, CIAO2B, and SDHB are associated with mitochondrial energy metabolism, so it is hypothesized that BspG may enter mitochondria to mediate the mitochondrial apoptotic pathway. In addition to this, the loss of BspG resulted in high levels of expression of the pro-inflammatory factors IL-1β and TNF-α, leading to an increase in their killing effect on host cells, which may be one of the reasons for the reduced survival of *B. abortus in vitro* and *in vivo* (Ma et al., 2022a). BspF can inhibit host cell apoptosis by attenuating the crotonylation modification of p53 and reducing the expression of p53, thus helping *Brucella* to survive for a long time (Lin et al., 2023). P53 plays an important role in the mitochondrial apoptotic pathway and the classical apoptotic pathway. In the mitochondrial pathway, P53 activates the expression of downstream genes such as Bax, PUMA, Noxa, and Bi, increases mitochondrial membrane permeability, and promotes the release of cytochrome C and ATP (Laptenko and Prives, 2006). In the classical apoptotic pathway, P53 binds to Apaf-1 and activates the caspase cascade reaction (Zhang et al., 2010).

### 5.5 Omp31 and Omp25 inhibit apoptosis

Omp31 and Omp25, the major outer membrane proteins of *Brucella*, can inhibit apoptosis. Omp31 is present in all *Brucella* species except *B. abortus* (Cloeckaert et al., 1996). In a macrophage model of *B. melitensis* infection, deletion of Omp31 resulted in elevated expression of TNF-α, Caspase-3, Caspase-8, Caspase-9, Bax, and Cytc, decreased expression of Bcl-2, and impaired *Brucella* survival (Zhang et al., 2016). It suggests that *Brucella* Omp31 has an inhibitory effect on the TNF-α-Caspase8-Caspase3 classical apoptotic pathway and the TNF-α-Cytc-Caspase9 mitochondrial apoptotic pathway. In BV-2 microglia, Omp31-induced autophagy achieves inhibition of TNF-α by negatively regulating the NF-κB P65 signaling pathway (Wang et al., 2021a). Omp31 or the autophagy inducer rapamycin inhibits the P65 signaling pathway proteins IκBα, P65 expression, and P65 phosphorylation, reduces the translocation of phosphor-P65 proteins to the nucleus, and ultimately decreases the level of TNF-α expression. The function that mitochondria play in the apoptotic pathway cannot be ignored, and when mitochondrial morphology is altered, such as by rupture, the mitochondrial apoptotic pathway is supposed to be disrupted. However, a study (Lobet et al., 2018) showed that mitochondria undergoing fragmentation affected neither TNF-α-induced apoptosis nor bacterial replication in *B. abortus*. The cause of the fragmentation that occurs in mitochondria is not fully understood and is not related to DRP1, a key effector of mitochondrial fission, but may be related to insufficient mitochondrial fusion. Like Omp31, Omp25 is the major outer membrane protein of *Brucella* that inhibits apoptosis of BV-2 microglia, but the exact mechanism of apoptosis is unknown (Ma et al., 2015).

## 6 Crosstalk clues between autophagy, inflammation, and apoptosis affected by *Brucella*

TNF-α regulates inflammatory responses and apoptosis. On the one hand, TNF-α amplifies and coordinates pro-inflammatory signaling. It binds to TNFR-1 to activate host resistance to *Brucella* and enhances the inflammatory response (Hop et al., 2017). On the other hand, TNF-α induces apoptosis. Inhibition of TNF-α production by *Brucella* virulence factors affects inflammatory responses and apoptosis. In addition, *Brucella*-induced autophagy seems to influence TNF-α production. Most of the inhibition of TNF-α by *Brucella* is related to the NF-κB signaling pathway. The NF-κB family is present in almost all cells and is involved in immune and inflammatory responses. In the cytoplasm, NF-κB dimers (e.g., NF-κB/P65) are usually in an inactivated state bound to IκB. When cells are subjected to various stimuli (e.g., TNF-α, interleukins), the IκB protein is degraded and the NF-κB dimer translocates to the nucleus to control the transcription of relevant genes (e.g., inflammatory factors and apoptosis genes) (Xiao, 2007). *Brucella* VceA inhibited the production of TNF-α and IL-1β, suggesting that VceA may influence the inflammatory response by inhibiting the production of pro-inflammatory cytokines (Zhang et al., 2019). In dendritic cells, *Brucella* Omp25 binds to SLAMF1 and inhibits NF-κB nuclear translocation thereby reducing the secretion of pro-inflammatory factors (TNF-α, IFN-γ, IL-6) (Degos et al., 2020). In macrophages, Omp25 suppressed LPS-induced TNF-α production. The relevant miRNAs targeted IRAK1 and TRAF6 proteins to prevent the NF-κB signaling pathway from being activated, thus reducing TNF-α production, which ultimately favored *Brucella* survival (Luo et al., 2017). *Brucella* Omp31 may impair the TNF-α-triggered classical apoptotic pathway and the mitochondrial apoptotic pathway. Autophagy was shown to control the NF-κB P65 signaling pathway through the degradation of regulatory proteins (Xiao, 2007). *B. melitensis* Omp31 inhibits the NF-κB P65 signaling pathway by inducing autophagy, thereby reducing TNF-α protein expression (Wang et al., 2021a).

The C-JUN N-terminal kinase (JNK) is associated with autophagy and apoptosis. Internalized *Brucella* activates the UPR sensor IRE1α, which activates IRE1α-associated kinases (including JNK and ASK1), and then drives the activation or assembly of autophagy proteins (e.g., ULK1, Beclin1, and ATG9a). The activities of these autophagy proteins contribute to cellular membrane remodeling to support the development of replicative niches (Pandey et al., 2018). Activated JNK can phosphorylate P53 and induce P53-dependent apoptosis. *B.suis* S2 inhibits activation of the JNK/P53 signaling pathway to suppress apoptosis in human microglia clone 3 cells by increasing the expression of CALR protein (Wang et al., 2021b).

AIR and ROS are associated with autophagy, inflammation, and apoptosis. The excessive ROS can be involved in the regulation of autophagy and programmed death by altering the activity of specific enzymes through redox reactions. Mitochondria are the main source of ROS. Verbeke et al. found that *B. abortus* induced mitophagy mediated by BNIP3L (Verbeke et al., 2023). TECPR1 is a protein that promotes the fusion of autophagosomes and lysosomes. AIR is an important domain of TECPR1. AIR binds to the ATG12-ATTG5 complex and then releases the Pleckstrin homology domain, which promotes autophagosome and lysosome fusion. *B. melitensis* 16M regulates the effects of the AIR domain on autophagy, inflammation, and apoptosis through the ROS signaling pathway. In *B. melitensis* 16M-infected macrophages, ROS induced apoptosis, inflammation, and autophagy, whereas AIR inhibited autophagosome maturation and autophagy initiation. Autophagy may negatively regulate the activation of inflammasomes and prevent inflammation (Li et al., 2016).

## 7 Conclusions

*Brucella* can manipulate autophagy, inflammation, and apoptosis for intracellular replication and survival. A range of virulence factors of *Brucella* can modulate autophagy, inflammation, and apoptosis, and play an important role in suppressing the immune response of the host cells (**Table 3**). In summary, *Brucella* has evolved some effective defense mechanisms to counteract host cell damage. However, the molecular and cellular mechanisms involved in the various survival methods of *Brucella* are not fully understood, and the functions of some of its virulence factors remain unclear. RicA, BtpA, BspB, BspF, and BspJ affect the biosynthesis of rBCV, but it is not clear whether other virulence factors have an effect on the biosynthesis of eBCV, rBCV, and aBCV. The specific mechanism by which VceA and BtpB inhibit autophagy is unclear. We have a preliminary understanding of the mechanisms by which VceC, BspJ, BspG, and BspF regulate apoptosis, but the mechanisms by which VceA and BtpB promote apoptosis remain unclear. The functions of certain virulence factors such as BspC, BspE, BPE005, BPE123, BPE043, and BPE275 are completely unknown. Related autophagy proteins are involved in intracellular replication in *Brucella*. However, the mechanism of interaction between *Brucella* and autophagy is not fully understood. After *Brucella* infection of the organism, host cells induce inflammatory responses through multiple pathways to defend against bacterial damage. We do not yet know what is the relationship between the impact of *Brucella* on inflammation and its chronic infection. Several effective and widely used animal vaccines have been produced, such as *B. abortus* S19/A19, *B. melitensis* Rev1, and *B. suis* S2. A vaccine against human brucellosis has not yet been developed; animal brucellosis vaccines are more diverse and effective, but also have drawbacks. Therefore, it is of great significance to have a comprehensive understanding of the pathogenesis of *Brucella*, which has a positive effect on the clinical development of drugs and facilitates the prevention and treatment of *Brucella* diseases.

**Table 3:** Evasion mechanisms of *Brucella* virulence factors.

Name	Type	Proposed function
VceA	T4SS effector	Inhibit autophagy and inflammatory factors (TNF-α and IL-1β) production. Promote apoptosis (Zhang et al., 2019).
VceC	T4SS effector	Induce inflammatory response through the IRE1 pathway (Keestra-Gounder et al., 2016). Induce unfolded protein response and regulate apoptosis (Byndloss et al., 2019; Zhi et al., 2019).
BtpA	T4SS effector	Inhibit inflammatory response and NF-κB activation through multiple pathways (Radhakrishnan et al., 2009; Sengupta et al., 2010; Chaudhary et al., 2012; Alaidarous et al., 2014; Jakka et al., 2017).
BtpB	T4SS effector	Inhibit autophagy and the inflammatory response of the TLR signaling pathway. Induce apoptosis (Li et al., 2022).
BspJ	T4SS effector	Inhibit apoptosis (Ma et al., 2020).
BspG	T4SS effector	Inhibit apoptosis (Ma et al., 2022a).
BspF	T4SS effector	Inhibit apoptosis (Lin et al., 2023).
LPS	Lipopolysaccharide	Inhibit recognition with the complement system. Evade TLR4 identification and attenuate the inflammatory response (Lapaque et al., 2006). Induce PMN cell death (Barquero-Calvo et al., 2015).
Omp16	Lipoprotein	Maintain the integrity and activity of the *Brucella* outer membrane (Zhi et al., 2020). Induce the production of inflammatory factors (Giambartolomei et al., 2004).
Omp19	Lipoprotein	Maintain the outer membrane properties of *Brucella* (Tibor et al., 2002). Evade the protease hydrolysis system (Pasquevich et al., 2019). Induce the production of inflammatory factors (Giambartolomei et al., 2004). Interact with TLR2 and inhibit MHC-II antigen presentation.
Omp25	Outer membrane protein	Inhibit apoptosis (Ma et al., 2015). Affect the autophagy mechanism (Jiao et al., 2020). Regulate miRNA to inhibit TNF-α production (Luo et al., 2017). Restrict NF-κB nuclear translocation in DC cells and inhibit secretion of pro-inflammatory factors (Degos et al., 2020). Inhibit the cGAS/STING signaling pathway and interfere with IFN-β production (Li et al., 2021). Inhibit LPS-induced IL-12 production (Cui et al., 2017).
Omp31	Outer membrane protein	Inhibit the classical apoptotic pathway and the mitochondrial apoptotic pathway (Zhang et al., 2016). Inhibit NF-κB p65 signal pathway and TNF-α expression (Wang et al., 2021a).
Flagella		Failure to recognize with TLR5 (Terwagne et al., 2013).
